# Comparison of Enoxaparin and Warfarin for Secondary Prevention of Cancer-Associated Stroke

**DOI:** 10.1155/2015/502089

**Published:** 2015-05-07

**Authors:** Hyemin Jang, Jung Jae Lee, Mi Ji Lee, Sookyung Ryoo, Chang Hyo Yoon, Gyeong-Moon Kim, Chin-Sang Chung, Kwang Ho Lee, Oh Young Bang, Suk Jae Kim

**Affiliations:** ^1^Department of Neurology, Samsung Medical Center, Sungkyunkwan University School of Medicine, Seoul 135-710, Republic of Korea; ^2^School of Health in Social Science, University of Edinburgh, 13/4 Lauriston Gardens, Edinburgh EH3 9HH, UK; ^3^Department of Neurology, Maria Sungmo Hospital, Seoul 150-038, Republic of Korea

## Abstract

*Background*. The aim of this study was to determine which anticoagulant is superior for secondary prevention of cancer-associated stroke, using changes in D-dimer levels as a biomarker for recurrent thromboembolic events.* Methods*. We conducted a retrospective, single center observational study including patients with cancer-associated stroke who were treated with either enoxaparin or warfarin. Blood samples for measuring the initial and follow-up D-dimer levels were collected at admission and a median of 8 days after admission, respectively. Multiple logistic regression analysis was conducted to evaluate the factors that influenced D-dimer levels after treatment.* Results*. Although the initial D-dimer levels did not differ between the two groups, the follow-up levels were dramatically decreased in patients treated with enoxaparin, while they did not change with use of warfarin (3.88 *μ*g/mL versus 17.42 *μ*g/mL, *p* = 0.026). On multiple logistic regression analysis, use of warfarin (OR 12.95; *p* = 0.001) and the presence of systemic metastasis (OR 18.73; *p* = 0.017) were independently associated with elevated D-dimer levels (≥10 *μ*g/mL) after treatment.* Conclusion*. In cancer-associated stroke patients, treatment with enoxaparin may be more effective than treatment with warfarin for lowering the D-dimer levels. Future prospective studies are warranted to show that enoxaparin is better than warfarin for secondary prevention in cancer-associated stroke.

## 1. Introduction

In recent years, there has been increasing interest in the association between cancer and cerebrovascular disease. However, the underlying pathophysiology of stroke in cancer patients is still not fully understood [1–3]. Recently, cancer-associated hypercoagulation has been proposed as the primary mechanism of stroke in these patients, particularly in those without vascular risk factors or conventional stroke etiologies [4–8]. Cancer-associated stroke has distinct characteristics including infarction of multiple vascular territories and markedly elevated D-dimer levels [7–13].

Keeping in mind the role of paraneoplastic hypercoagulability in the development of thrombosis in the setting of malignancy [1], strategies for prevention of recurrent embolism in patients with cancer-associated stroke should theoretically focus on correction of the coagulopathy using anticoagulants. Based on the findings of large clinical trials, low-molecular-weight heparin is the preferred agent for treatment of venous thromboembolism in patients with cancer [14, 15]. However, there is not much data regarding optimal medications for secondary prevention of cancer-associated stroke.

In this study, we aimed to determine whether enoxaparin is superior to warfarin for prevention of recurrent stroke using changes in the D-dimer level, which is a known biomarker for predicting cancer-associated thrombotic events [12, 16, 17].

## 2. Materials and Methods

### 2.1. Patient Selection and Initial Workup

We analyzed data from consecutive patients with cancer-associated stroke who presented within 7 days of symptom onset between July 2006 and December 2012. Cancer-associated stroke was identified when the patient had (1) active cancer (diagnosis of cancer within 6 months of stroke onset, any treatment for cancer within the previous 6 months, or recurrent or metastatic cancer) [18] and (2) ischemic stroke which could not be explained by conventional stroke mechanisms including large artery atherosclerosis, cardioembolism, lacunar infarction, or other etiologies (e.g., dissection) [19]. Among the patients that met these criteria, we included subjects who were treated with either enoxaparin or warfarin for prevention of recurrent stroke. Patients who had primary intracranial malignancy, an incomplete workup for stroke etiology (either vascular or cardiologic studies), or a history of recent surgery, myocardial infarction, or any signs of infectious or immunological diseases which may influence plasma D-dimer levels were excluded. Patients with stroke suspected to be caused by the tumor itself (i.e., tumor emboli) or cancer treatment (i.e., chemotherapy-induced stroke) were also excluded. The Institutional Review Board in Samsung Medical Center waived the need for written consent from the patients and approved this study. The records of the patients were anonymized prior to analysis.

Demographic and clinical data including age, sex, prestroke medications, and vascular risk factors were collected at the time of admission. The type of primary cancer, histopathologic findings, and presence/absence of systemic metastasis were also recorded. Blood samples were drawn at the time of admission and were analyzed with a standard battery of biochemical and hematological tests. All patients underwent brain MRI, vascular studies, 12-lead electrocardiography, Holter and/or telemetry monitoring, and noninvasive transthoracic echocardiography, which was preferable in these critically ill patients with advanced cancer. The patterns of acute stroke on diffusion-weighted imaging (DWI) were reviewed and classified as single/multiple lesions involving one vascular territory or multiple lesions involving multiple vascular territories by two independent readers (K. S. J. and J. H. M.). Both readers were blinded to the treatment groups and D-dimer levels, and discordance in classification was resolved by consensus.

### 2.2. Treatment and Outcomes

All subjects received either twice-daily subcutaneous injections of enoxaparin (1.0 mg/kg) or oral warfarin with a target international normalized ratio (INR) between 2.0 and 3.0. The warfarin group was initially treated with heparin (intravenous unfractionated heparin or enoxaparin) until reaching target levels of INR. We reviewed medical records to identify major bleeding events or recurrent strokes during the course of treatment. A bleeding event was considered major if it caused a fall in hemoglobin of 2 g/dL or more, transfusion of at least 2 units of packed red blood cells, or intracranial hemorrhage.

At the start of the study period, most patients were treated with warfarin without measuring the D-dimer level after initiating anticoagulation, since the role of monitoring D-dimer levels for therapeutic effects had not yet been established. However, after one patient with recurrent stroke was found to have increasing D-dimer levels after switching from enoxaparin to warfarin, patients were subsequently given either enoxaparin or warfarin depending on the preference of the individual physician with follow-up measurements of D-dimer levels as a surrogate biomarker of recurrent stroke [5, 11, 12]. The attending stroke physicians were sequentially determined depending on the date of admission. Blood samples for follow-up D-dimer levels were collected at a median of 8 days (interquartile range 6–11 days) after admission. The D-dimer was measured by immunoturbidimetry on a STA-R automated analyzer (Diagnostica Stago, Asnieres, France) (reference values in our laboratory ≤0.5 mg/mL).

### 2.3. Statistical Analyses

Statistical analyses were performed using a commercially available software package (PASW version 18.0; SPSS Inc., Chicago, IL, USA). All data are presented as medians (25th–75th percentile) or numbers (percentages). The Shapiro-Wilk test was used to confirm a normal distribution. Since the distributions of all variables were not normal (*p* < 0.05), Mann-Whitney *U* tests were conducted to compare continuous variables between groups. Pearson's chi-square or Fisher's exact tests were used to compare categorical variables. We used the Bonferroni correction to account for multiple comparisons. Rates of recurrent stroke and major bleeding events were computed by the Kaplan-Meier method and compared using a two-sided log-rank test. In addition, multiple logistic regression analysis was conducted to evaluate the independent contribution of factors that influenced D-dimer levels after treatment. Variables that were significant at *p* < 0.2 on univariable analyses were considered explanatory variables and were entered together into a multivariable model. Results are reported as odds ratios (OR) with 95% confidence intervals (CI). Moreover, a multiple linear regression model was also performed to identify factors associated with the change in D-dimer levels between baseline and follow-up measurements. A *p* value of <0.05 was considered to be statistically significant.

## 3. Results

### 3.1. Patient Characteristics

Of the 104 patients with cancer-associated stroke who were admitted during the study period, 79 patients treated with either enoxaparin or warfarin were included. Baseline characteristics of the patients are presented in Table 1. Age, sex, and the presence of vascular risk factors did not differ significantly between the two groups. Initial laboratory findings before treatment including D-dimer levels were also comparable between groups. Multiple lesions involving multiple vascular territories on DWI were most frequently encountered in both groups.

In terms of cancer profiles, both groups had similar characteristics including location of the primary cancer and type of histopathology. However, the presence of systemic metastasis at the time of stroke was more prevalent in patients treated with enoxaparin than warfarin (93.1% versus 62.0%; *p* = 0.003).

### 3.2. Recurrent Stroke and Major Bleeding Events

During the mean follow-up period of 4.9 months, recurrent strokes were noted in only 1 (3.4%) patient treated with enoxaparin and in 8 (16.0%) patients treated with warfarin. However, the difference in stroke recurrence between the two groups did not reach statistical significance primarily due to small sample size (*p* = 0.249 by the log-rank test). Detailed features of patients with recurrent stroke are shown in Table 2. Time to recurrence was usually within 2 months of the initial event, and most patients had D-dimer levels that were >10 *μ*g/mL and an INR above the lower limit (2.0). The incidence of major bleeding events was similar between the two groups (6.9% for enoxaparin versus 10.0% for warfarin; *p* = 0.960 by log-rank test).

### 3.3. Changes in D-Dimer Levels after Anticoagulation

**Figure 1:**
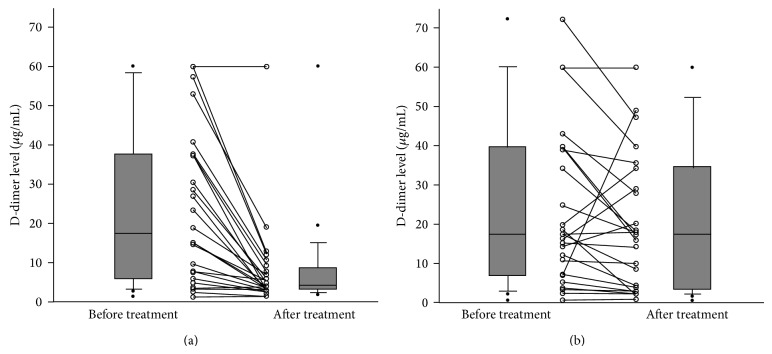
Changes in D-dimer levels after treatment: (a) enoxaparin; (b) warfarin.

Follow-up D-dimer levels were available for 52 of 79 patients (26 in each group). The median time from baseline to follow-up measurement was not significantly different between groups (8 days (5–12 days) for enoxaparin versus 8 days (6–9 days) for warfarin; *p* = 0.686).  Figure 1 illustrates the changes in D-dimer levels after treatment. The initial concentrations were comparable for patients treated with enoxaparin and warfarin (17.06 *μ*g/mL (5.62–37.48 *μ*g/mL) for enoxaparin versus 17.78 *μ*g/mL (9.57–39.54 *μ*g/mL) for warfarin; *p* > 0.999). However, the follow-up levels were dramatically decreased in patients treated with enoxaparin, while they did not change significantly in those treated with warfarin (3.88 *μ*g/mL (3.01–8.12 *μ*g/mL) for enoxaparin versus 17.42 *μ*g/mL (3.34–34.38 *μ*g/mL) for warfarin; *p* = 0.026).

Since 7 of 9 patients who had a recurrent stroke had D-dimer levels of ≥10 *μ*g/mL at the time of recurrence, we divided the 52 patients into two groups according to a cutoff value of 10 *μ*g/mL after treatment. As shown in Table 3, treatment with warfarin, systemic metastasis, and adenocarcinoma were more prevalent in patients with follow-up D-dimer levels ≥10 *μ*g/mL. On multiple logistic regression analysis, anticoagulation using warfarin (adjusted OR 12.95; 95% CI, 2.89–57.94; *p* = 0.001) and the presence of systemic metastasis (adjusted OR 18.73; 95% CI, 1.69–207.48; *p* = 0.017) were independently associated with D-dimer levels of ≥10 *μ*g/mL (Table 4).

Sensitivity analysis was subsequently performed using different cutoff values for D-dimer levels after anticoagulation, since the designation of ≥10 *μ*g/mL was somewhat arbitrary. Oral anticoagulation and metastasis were also independently associated with follow-up D-dimer levels of ≥5 and ≥15 *μ*g/mL. In addition, multiple linear regression analysis demonstrated that treatment with enoxaparin was the only factor that was independently associated with a decline in D-dimer levels during treatment (*p* = 0.020).

## 4. Discussion

In patients with cancer, thromboembolic complications negatively affect quality of life and increase the risk of death. Our results demonstrate that the risk of recurrence among patients with cancer-associated stroke may be lower with use of enoxaparin rather than oral warfarin for anticoagulation therapy. In addition, we did not find a significant difference in the rates of major bleeding events between the groups, which is in agreement with previous reports [18, 20]. Although it is known that low-molecular-weight heparin is superior to warfarin for secondary prophylaxis of venous thromboembolism in patients with active cancer [18], there is a paucity of data regarding the optimal medication in patients with cancer-associated stroke. To the best of our knowledge, this is the first study to compare the efficacy and safety of different types of anticoagulants for preventing recurrent stroke in this population.

On a biochemical level, tissue factor is an important component that triggers thromboembolic events in cancer patients [21]. Accordingly, inhibition of the extrinsic coagulation pathway that is initiated by tissue factor should be the primary target for management of cancer-associated thromboembolism. Heparin (both unfractionated and low-molecular-weight), but not warfarin, can release tissue factor pathway inhibitor (TFPI) which binds to the complex of tissue factor, factor VIIa, and factor X, ultimately blocking the production of factor Xa [22]. This may explain the superiority of heparin over warfarin in reducing recurrent thromboembolic complications in patients with active cancer and corroborate the results of our study [15, 18].

The D-dimer is a degradation product of cross-linked fibrin which reflects activation of the coagulation system; thus elevated D-dimer levels are suggestive of a hypercoagulable state [23, 24]. In addition, D-dimer levels are useful for prognostication as well as judging of the effect of anticoagulation treatment in patients with deep vein thrombosis, pulmonary embolism, and cancer-associated stroke [5, 25–27]. Since the life expectancy of our study population is shorter than average due to the presence of advanced cancer, it is difficult to achieve adequate follow-up until having recurrent embolic events. For this reason, we used the D-dimer level as a biomarker for monitoring treatment efficacy and predicting stroke recurrence.

There are several limitations to our study. First, because of its nature as a nonrandomized retrospective study, measurement of follow-up D-dimer levels was completed in different portion of the patients in each group. In the initial period of this study, D-dimer levels over treatment were not checked routinely due to lack of knowledge about their role as a biomarker. Because most physicians chose warfarin as maintenance anticoagulation at that time, many patients in warfarin group were excluded because of missed data. Moreover, treatment choice was dependent on the judgment of individual physicians. Also, patients were not routinely screened for venous thromboembolism which is an important cause of elevated D-dimer. Therefore, we are planning to perform a prospective, randomized trial to confirm our observational study results. Second, our results are promising, but they are restricted by small sample size. Thus, additional study in a large number of patients with cancer-associated stroke is required. Finally, we defined cancer-associated stroke by excluding patients with conventional stroke mechanisms. To resolve this issue, identification of a biomarker specific to cancer-associated stroke and associated with pathophysiology is needed.

In conclusion, enoxaparin may be more effective than warfarin for lowering the D-dimer levels in patients with cancer-associated stroke. Future prospective studies are warranted to show that enoxaparin is better than warfarin for secondary prevention in cancer-associated stroke itself.

## Figures and Tables

**Table 1:** Baseline characteristics.

	Enoxaparin	Warfarin	*p*
	(*N* = 29)	(*N* = 50)	
Male sex	18 (62.1%)	24 (48.0%)	0.227
Age, years	64 (53–67)	66 (60–72)	0.099
Vascular risk factors			
Hypertension	6 (20.7%)	21 (42.0%)	0.054
Diabetes	7 (24.1%)	10 (20.0%)	0.666
Hyperlipidemia	1 (3.4%)	6 (12.0%)	0.252
Current smoker	7 (24.1%)	10 (20.0%)	0.666
Coronary artery disease	1 (3.4%)	1 (2.0%)	>0.999
Laboratory findings on admission			
Platelet, ×10^3^/*μ*L	149 (89–249)	151 (108–229)	0.955
D-dimer, *μ*g/mL	15.08 (4.34–37.32)	11.35 (2.84–37.25)	0.413
Fibrinogen, mg/dL	377 (294–500)	275 (184–432)	0.062
DWI^∗^ patterns			0.612
Single vascular territory	5 (17.2%)	11 (22.0%)	
Single lesion	2	4	
Multiple lesions	3	7	
Multiple vascular territories	24 (82.8%)	39 (78.0%)	
Prestroke medication			
Antiplatelet agents	1 (3.4%)	8 (16.0%)	0.143
Anticoagulants	2 (6.9%)	4 (8.0%)	>0.999

Cancer profiles			
Primary cancer type			0.229
Lung	11 (37.9%)	27 (54.0%)	
Gastrointestinal	6 (20.7%)	6 (12.0%)	
Hepatobiliary	5 (17.2%)	10 (20.0%)	
Breast-gynecologic	5 (17.2%)	2 (4.0%)	
Other	2 (6.9%)	5 (10.0%)	
Systemic metastasis	27 (93.1%)	31 (62.0%)	0.003
Adenocarcinoma	19 (65.5%)	35 (71.4%)	0.585

^∗^DWI: diffusion-weighted imaging.

**Table 2:** Summary of detailed characteristics of subjects with recurrent stroke.

Number	Sex	Age	Treatment	D-dimer level (*μ*g/mL) on admission	D-dimer level (*μ*g/mL) at the time of recurrence	INR^∗^ at the time of recurrence	Time to recurrence (months)	Primary cancer type	Adenocarcinoma	Systemic metastasis
1	Female	55	Warfarin	19.54	47.80	3.04	1.13	Lung	Yes	Yes
2	Male	72	Warfarin	1.91	2.96	1.48	0.80	Lung	Yes	No
3	Female	59	Warfarin	72.40	47.20	2.60	0.83	CBD	Yes	Yes
4	Male	61	Warfarin	60.00	39.72	2.62	0.46	Colon	Yes	Yes
5	Female	60	Warfarin	18.36	10.30	1.39	8.30	Lung	Yes	Yes
6	Female	51	Warfarin	11.74	57.93	5.51	19.63	Lung	Yes	Yes
7	Female	48	Warfarin	4.83	0.34	3.26	1.90	Lung	Yes	Yes
8	Female	75	Warfarin	14.87	14.74	2.06	0.37	Pancreas	Yes	Yes
9	Male	67	Enoxaparin	60.00	60.00	N/A^†^	0.43	CBD^‡^	Yes	Yes

^∗^INR, international normalized ratio; ^†^N/A: not applicable; ^‡^CBD: common bile duct.

**Table 3:** Factors associated with high D-dimer levels.

	D-dimer ≥10 *μ*g/mL after treatment		
	No	Yes	*p* ^∗^
	(*n* = 31)	(*n* = 21)	
Male sex	18 (58.1%)	10 (47.6%)	0.458
Age, years	63 (55–71)	63 (55–70)	0.970
DWI patterns			0.724
Single vascular territory	6 (19.4%)	3 (14.3%)	
Single lesion	3	2	
Multiple lesions	3	1	
Multiple vascular territories	25 (80.6%)	18 (85.7%)	
Treatment			0.002
Enoxaparin	21 (67.7%)	5 (23.8%)	
Warfarin	10 (32.3%)	16 (72.6%)	
Cancer profiles			
Primary cancer type			0.586
Lung	15 (48.4%)	12 (57.1%)	
Gastrointestinal	4 (12.9%)	3 (14.3%)	
Hepatobiliary	5 (16.1%)	5 (23.8%)	
Breast-gynecologic	4 (12.9%)	1 (4.8%)	
Other	3 (9.7%)	0 (0%)	
Systemic metastasis	23 (74.2%)	20 (95.2%)	0.067
Adenocarcinoma	19 (61.3%)	17 (85.0%)	0.070

^∗^Other factors including vascular risk factors, premedications, and laboratory findings (platelet and fibrinogen) did not differ between the two groups (*p* > 0.2).

**Table 4:** Multiple logistic regression analysis: independent predictors of D-dimer levels ≥10 *μ*g/mL.

	Estimated OR^∗^		
	Univariable	Multivariable	*p*
	(95% CI^†^)	(95% CI)	
Treatment			
Enoxaparin	Reference	Reference	
Warfarin	6.72 (1.92–23.58)	12.95 (2.89–57.94)	0.001
Systemic metastasis	6.96 (0.80–60.53)	18.73 (1.69–207.48)	0.017
Adenocarcinoma	3.58 (0.86–14.87)	2.41 (0.43–13.44)	0.314

^∗^OR: odds ratio; ^†^CI: confidence interval.
